# *PTGER4* Expression-Modulating Polymorphisms in the 5p13.1 Region Predispose to Crohn's Disease and Affect NF-κB and XBP1 Binding Sites

**DOI:** 10.1371/journal.pone.0052873

**Published:** 2012-12-27

**Authors:** Jürgen Glas, Julia Seiderer, Darina Czamara, Giulia Pasciuto, Julia Diegelmann, Martin Wetzke, Torsten Olszak, Christiane Wolf, Bertram Müller-Myhsok, Tobias Balschun, Jean-Paul Achkar, M. Ilyas Kamboh, Andre Franke, Richard H. Duerr, Stephan Brand

**Affiliations:** 1 Department of Medicine II - Grosshadern, University of Munich, Munich, Germany; 2 Department of Preventive Dentistry and Periodontology, University of Munich, Munich, Germany; 3 Department of Human Genetics, Rheinisch-Westfälische Technische Hochschule (RWTH) Aachen, Aachen, Germany; 4 Max-Planck-Institute of Psychiatry, Munich, Germany; 5 Center for Pediatrics, Hannover Medical School, Hannover, Germany; 6 Division of Gastroenterology, Brigham & Women's Hospital, Harvard Medical School, Boston, United States of America; 7 Institute of Clinical Molecular Biology, Christian-Albrechts-University, Kiel, Germany; 8 Department of Pathobiology, Lerner Research Institute, Cleveland Clinic, Cleveland, Ohio, United States of America; 9 Department of Gastroenterology and Hepatology, Digestive Disease Institute, Cleveland Clinic, Cleveland, Ohio, United States of America; 10 Department of Human Genetics, Graduate School of Public Health, University of Pittsburgh, Pittsburgh, Pennsylvania, United States of America; 11 Division of Gastroenterology, Hepatology and Nutrition, School of Medicine, University of Pittsburgh, Pittsburgh, Pennsylvania, United States of America; Kunming Institute of Zoology, Chinese Academy of Sciences, China

## Abstract

**Background:**

Genome-wide association studies identified a PTGER4 expression-modulating region on chromosome *5p13.1* as Crohn's disease (CD) susceptibility region. The study aim was to test this association in a large cohort of patients with inflammatory bowel disease (IBD) and to elucidate genotypic and phenotypic interactions with other IBD genes.

**Methodology/Principal Findings:**

A total of 7073 patients and controls were genotyped: 844 CD and 471 patients with ulcerative colitis and 1488 controls were analyzed for the single nucleotide polymorphisms (SNPs) rs4495224 and rs7720838 on chromosome *5p13.1*. The study included two replication cohorts of North American (CD: n = 684; controls: n = 1440) and of German origin (CD: n = 1098; controls: n = 1048). Genotype-phenotype, epistasis and transcription factor binding analyses were performed. In the discovery cohort, an association of rs4495224 (p = 4.10×10^−5^; 0.76 [0.67–0.87]) and of rs7720838 (p = 6.91×10^−4^; 0.81 [0.71–0.91]) with susceptibility to CD was demonstrated. These associations were confirmed in both replication cohorts. *In silico* analysis predicted rs4495224 and rs7720838 as essential parts of binding sites for the transcription factors NF-κB and XBP1 with higher binding scores for carriers of the CD risk alleles, providing an explanation of how these SNPs might contribute to increased PTGER4 expression. There was no association of the *PTGER4* SNPs with IBD phenotypes. Epistasis detected between *5p13.1* and *ATG16L1* for CD susceptibility in the discovery cohort (p = 5.99×10^−7^ for rs7720838 and rs2241880) could not be replicated in both replication cohorts arguing against a major role of this gene-gene interaction in the susceptibility to CD.

**Conclusions/Significance:**

We confirmed *5p13.1* as a major CD susceptibility locus and demonstrate by *in silico* analysis rs4495224 and rs7720838 as part of binding sites for NF-κB and XBP1. Further functional studies are necessary to confirm the results of our *in silico* analysis and to analyze if changes in PTGER4 expression modulate CD susceptibility.

## Introduction

Crohn's disease (CD) and ulcerative colitis (UC) are chronic inflammatory bowel diseases (IBD) characterized by a complex molecular pathogenesis resulting in an exaggerated immune response and mucosal destruction [1]–[3]. Recent insights in the interaction of various susceptibility genes with intestinal bacteria have substantially helped to unravel the pathogenesis of IBD [2], [4]. Since the identification of *NOD2* as the first susceptibility gene for CD in 2001 [5], [6], various studies including genome-wide association studies (GWAS) based on high-density SNP (single nucleotide polymorphism) arrays have identified CD-associated genetic variants of proteins involved in immune response, autophagy or bacterial recognition, such as *IL23R* [7], [8], *ATG16L1* [9]–[11], and *IRGM* [12]. In addition, genotype-phenotype analysis by us and others also demonstrated significant associations for certain gene variants with particular CD phenotypes [13]–[20].

In 2007, a GWAS analyzing more than 318,000 SNPs identified a 250 kb region on chromosome *5p13.1* contributing to CD susceptibility [21]. The disease-associated alleles were found to correlate with expression levels of the prostaglandin receptor EP4, which binds prostaglandin E2 (PGE2) and is encoded by *PTGER4*, the gene located closest to the associated region [21]. Since *Ptger4^−/−^* mice develop severe dextran sodium sulphate (DSS)-induced colitis while treatment with EP4-selective agonists has protective effects against colitis through enhancement of epithelium survival and regeneration, *PTGER4* represents an attractive IBD candidate gene [22]–[24]. Prostaglandins are arachidonic acid metabolites produced by the action of the enzymes cyclooxygenase (COX)-1 and -2 and play a crucial role in the regulation of gastrointestinal homeostasis and IBD pathogenesis [25]–[27]. The novel *5p13.1* CD susceptibility locus in proximity of *PTGER4* was also replicated by a recent genome-wide association study [28].

Moreover, the data on the disease-modifying effect of this region on UC is very limited so far. In a recent GWAS of UC [29], a significant association between rs4613763 variant in the *5p13* region and UC has been reported. To analyze the effect of SNPs in the *5p13.1* region on IBD susceptibility in the German population, a large study was initiated and genomic DNA of 2803 individuals was genotyped for the two SNPs rs4495224 and rs7720838, identified in the initial study by Libioulle and co-workers as CD susceptibility locus [21]. Although other SNPs in the study by Libioulle et al. [21] showed moderately stronger association with CD, these two SNPs were selected for genotyping since they were both strongly associated with CD and showed the most significant effect on PTGER4 expression in that GWAS. In addition, the detailed phenotypic consequences of these gene variants in CD and UC were here analyzed for the first time. Moreover, we aimed to identify whether rs4495224 and rs7720838 are part of potential binding sites for transcription factors that might influence PTGER4 expression. As *5p13.1* may also interact with other IBD susceptibility genes, a detailed analysis for potential epistasis with the previously identified major CD susceptibility gene variants (in *NOD2*, *IL23R*, *ATG16L1* and in *SLC22A4/5* in the IBD5 region) was performed.

## Methods

### Ethics statement

This study was approved by the Ethics committee of the Medical Faculty of Ludwig-Maximilians-University Munich (discovery cohort), the University Hospital of the Christian-Albrechts-University Kiel (German replication cohort), the Cleveland Clinic and the University of Pittsburgh (North American replication cohort). Written, informed consent was obtained from all patients prior to genotyping and inclusion into the study. In the case of minors, the consent was provided by the parents. The study protocol adhered to the ethical principles for medical research involving human subjects of the Helsinki Declaration (as described in detail under: http://www.wma.net/en/30publications/10policies/b3/index.html).

### Study population and disease phenotype analysis

Overall, the German discovery study population (n = 2803) included 1315 IBD patients of Caucasian origin consisting of 844 patients with CD, 471 patients with UC, and 1488 healthy, unrelated controls. In order to replicate the association of chromosome *5p13.1* SNPs, a U.S. American Caucasian CD cohort (CD: n = 684; controls: n = 1440) from the University of Pittsburgh and the Cleveland Clinic and an additional German replication cohort from the University Hospital of Schleswig-Holstein at Kiel (CD: n = 1098; controls: n = 1048) were investigated. The diagnosis of CD or UC was based on established guidelines including endoscopic, radiological, and histopathological criteria [30]. Patients with CD were assessed according to the Montreal classification [31] analyzing age at diagnosis (A), location (L), and behavior (B) of disease. In patients with UC, anatomic location was also assessed in accordance to the Montreal classification, using the criteria ulcerative proctitis (E1), left-sided UC (distal UC; E2), and extensive UC (pancolitis; E3). Patients with indeterminate colitis were excluded from the study. Phenotypic characteristics were collected blind to the results of the genotypic data and included demographic and clinical parameters (behavior and anatomic location of IBD, disease-related complications, surgical or immunosuppressive therapy) which were recorded by analysis of patient charts and a detailed questionnaire including an interview at time of enrolment. The demographic characteristics of the IBD study population are summarized in Table 1.

**Table 1:** Demographic characteristics of the German IBD discovery study population.

	Crohn's disease	Ulcerative colitis	Controls
	n = 844	n = 471	n = 1488
**Gender**			
Male (%)	51.4%	52.1%	62.9%
**Age** (yrs)			
Mean ± SD	39.5±13.1	41.8±14.5	45.9±10.7
Range	10–80	7–85	18–71
**Body mass index**			
Mean ± SD	23.1±4.2	23.9±4.2	
Range	13–40	15–41	
**Age at diagnosis** (yrs)			
Mean ± SD	27.7±11.7	32.0±13.4	
Range	1–78	9–81	
**Disease duration** (yrs)			
Mean ± SD	11.8±8.5	10.4±7.8	
Range	0–44	1–40	
**Positive family**			
**history of IBD (%)**	16.0%	16.1%	

### DNA extraction and genotyping of SNPs in the 5p13.1 region

Genomic DNA was isolated from peripheral blood leukocytes by standard procedures using the DNA blood mini kit from Qiagen (Hilden, Germany). The SNPs rs4495224 and rs7720838 on chromosome *5p13.1*, for which significant associations with CD were found in a previous study [21], were genotyped by PCR and melting curve analysis using a pair of fluorescence resonance energy transfer (FRET) probes in a LightCycler 480 system (Roche Diagnostics, Mannheim, Germany), using a similar methodology as described previously [8], [11]. The results of melting curve analysis were confirmed by analyzing samples representing all possible genotypes using sequence analysis. All sequences of primers and FRET probes and primer annealing temperatures used for genotyping and for sequence analysis are given in Tables S1 and S2.

In the U.S. American cohort, SNPs rs4532399, rs11955354, rs11957215, rs7720838, and rs10440635 in the *5p13.1* region were genotyped using the Human Omni1-Quad chip (Illumina, Inc., San Diego, CA). In the German replication cohort, SNPs rs7720838 and rs10941508 (surrogate marker for rs4495224) in the *5p13.1* region were genotyped using the SNPlex technology (Applied Biosystems) in an automated laboratory setup and all process data were written to and administered by a database-driven laboratory information management system.

### Genotyping of variants in NOD2, IL23R, ATG16L1, and SLC22A4/5

From previous studies, the genotypes of CD-associated gene variants in *NOD2* [16], [17], [19], *IL23R* [8], *ATG16L1* [11] and *SLC22A4/5* [32] were available for the German discovery study cohort. Genotyping of the *NOD2* variants p.Arg702Trp (rs2066844), p.Gly908Arg (rs2066845), and p.Leu1007fsX1008 (rs2066847) as well as analysis of the 1672 C→T SNP in *SLC22A4* (rs1050152) encoding OCTN1 and the –207 G→C SNP (rs2631367) in *SLC22A5* encoding OCTN2 were performed by PCR and restriction fragment length polymorphism analysis as described previously [32]. The primer sequences, the restriction enzymes used and the resulting fragment lengths are given in Table S3. The 10 CD-associated *IL23R* SNPs (rs1004819, rs7517847, rs10489629, rs2201841, rs11465804, rs11209026 = p.Arg381Gln, rs1343151, rs10889677, rs11209032, rs1495965) described by Duerr and co-workers [7] and nine *ATG16L1* SNPs (rs13412102, rs12471449, rs6431660, rs1441090, rs2289472, rs2241880 ( = p.Thr300Ala), rs2241879, rs3792106, rs4663396) described by Hampe and co-workers [9] were genotyped by PCR and melting curve analysis as described previously [8], [11]. All sequences of primers and FRET probes and primer annealing temperatures used for genotyping and for sequence analysis are given in Tables S4 and S5.

In the U.S. American cohort, *ATG16L1* SNPs rs13412102, rs3828309, rs2289474, rs2241880 ( = p.Thr300Ala), and rs2241879 were genotyped using the Human Omni1-Quad chip (Illumina, Inc.). In the German replication cohort, *ATG16L1* SNPs rs13412102, rs12471449, rs6431660, rs1441090, rs2289472, rs2241880 ( = p.Thr300Ala), rs2241879, rs3792106, rs4663396 were genotyped using the SNPlex technology (Applied Biosystems). Since genotyping was performed in the IBD centers in which the blood samples were centrally collected, each IBD center used its own “in house” genotyping protocol (Munich: PCR and melting curve analysis using a pair of FRET probes; Kiel: SNPlex technology (Applied Biosystems); Pittsburgh: Human Omni1-Quad chip (Illumina, Inc.)). Given the differences in the genotyping platforms, not all SNPs were identical; therefore, surrogate markers with high linkage disequilibrium were used where appropriate.

### In silico analysis of transcription factor binding sites

SNPs rs4495224 and rs7720838 were analyzed for potential human transcription factor binding sites applying the online tool TFSEARCH which is based on the TRANSFAC database [33]. Transcription factors with predicted binding scores of ≥75 for each allele were included in the analysis (max. score = 100). For each SNP, major and minor alleles including the flanking 15 nucleotides upstream (5′) and downstream (3′) were analyzed.

### Statistical analyses

Data were evaluated by using the SPSS 13.0 software (SPSS Inc., Chicago, IL, U.S.A.) and R-2.4.1. (http://cran.r-project.org). Each genetic marker was tested for Hardy-Weinberg equilibrium in the control population. Fisher's exact test was used for comparison between categorical variables, while Student's t test was applied for quantitative variables. Single-marker allelic tests were performed with Pearson's χ^2^ test. All tests were two-tailed, considering p-values<0.05 as significant. Odds ratios were calculated for the minor allele at each SNP. For multiple comparisons, Bonferroni correction was applied where indicated. Interactions between different polymorphisms were tested using the –epistasis option provided in PLINK (http://pngu.mgh.harvard.edu/~purcell/plink/).

## Results

### The SNPs rs4495224 and rs7720838 in the 5p13.1 region are significantly associated with Crohn's disease

In all three subgroups (CD, UC, and controls) of the German discovery study cohort, the allele frequencies of the SNPs rs4495224 and rs7720838 were in accordance with the predicted Hardy-Weinberg equilibrium and are summarized in Table 2. Similar to the results of the study of Libioulle et al. (D′ = 0.84) [21], both *PTGER4* expression-modulating SNPs were in linkage disequilibrium (controls: D′ = 0.843; CD: D′ = 0.795; UC: D′ = 0.871; all cohorts: D′ = 0.836).

**Table 2:** Allele frequencies of the SNPs rs4495224 and rs7720838 in German discovery population of patients with Crohn's disease and ulcerative colitis and controls.

Gene	Minor	Crohn's disease			Ulcerative colitis			Controls
marker	allele	n = 844			n = 471			n = 1488
		MAF	p value	OR [95% CI]	MAF	p value	OR [95% CI]	MAF
rs4495224	C	0.28	4.10×10^−5^	0.76 [0.67–0.87]	0.35	3.17×10^−1^	1.08 [0.93–1.26]	0.34
rs7720838	G	0.38	6.91×10^−4^	0.81 [0.71–0.91]	0.45	2.75×10^−1^	1.09 [0.94–1.26]	0.43

Note: Minor allele frequencies (MAF), allelic test p-values, and odds ratios (OR, shown for the minor allele) with 95% confidence intervals (CI) are shown.

Overall, significant differences in the frequencies of rs4495224 and rs7720838 were observed in CD patients compared to healthy controls (Table 2), identifying SNP rs4495224 and rs7720838 as significantly CD-associated genetic variants. In the CD group, the frequency of the rarer C allele of the rs4495224 polymorphism was 0.28, whereas in the controls it was 0.34 (p = 4.10×10^−5^, OR 0.76 [0.67–0.87]). The frequencies of the minor G allele of the rs7720838 polymorphism were 0.375 in CD and 0.43 in the controls (p = 6.91×10^−4^, OR 0.81 [0.71–0.91]), suggesting a protective effect of the minor allele in CD. In contrast, no associations were observed in UC patients. In UC, the frequencies of the rs4495224 C allele and of the rs7720838 G allele were 0.35 (p = 3.17×10^−1^, OR 1.08 [0.93–1.26]) and 0.45 (p = 2.75×10^−1^, OR 1.09 [0.94–1.26]), respectively. However, the lack of association with UC could be related to a lack of power. In a power analysis using the Genetics Power Calculator (http://pngu.mgh.harvard.edu/~purcell/gpc/), we used the settings unselected controls and, a minor allele frequency of 43%. Considering our sample size of 471 UC cases and 1488 controls, our study had 25% power for detecting differences in the minor allele frequencies between cases (UC) and controls corresponding to an OR of 1.10.

In the North American CD cohort, all 5 SNPs investigated in the *PTGER4* region were strongly associated with CD susceptibility (Table 3). The SNPs rs4532399, rs11955354 and rs11957215, which were in nearly complete linkage disequilibrium according to the data of the Human HapMap project, were used as surrogate markers for SNP rs4495224. The minor allele frequencies of all these three SNPs were 0.28 in the North American CD cohort and 0.35 the in the North American control population (rs4532399: p = 3.07×10^−6^; 0.72 [0.62–0.82], rs11955354: p = 5.45×10^−6^; 0.72 [0.63–0.83], rs11957215: p = 7.08×10^−6^; 0.72 [0.63–0.83]). The frequencies of the minor G allele of the SNP rs7720838 were 0.375 in CD and 0.45 in the controls (p = 2.19×10^−7^; 0.71 [0.62–0.81]). For the SNP rs10440635, which is a surrogate marker for rs7720838, the minor allele frequencies were 0.36 in the CD cohort and 0.45 in the control population (p = 8.60×10^−8^; 0.70 [0.61–0.80]).

**Table 3:** Allele frequencies of the SNPs within the *5p13.1* region in North American replication cohort.

Gene marker	Gene/region	Minor allele	Crohn's disease			Controls
			n = 684			n = 1440
			MAF	p value	OR [95% CI]	MAF
rs4532399*	*5p13.1*	A	0.28	3.07×10^−6^	0.72 [0.62–0.82]	0.35
rs11955354*	*5p13.1*	A	0.28	5.45×10^−6^	0.72 [0.63–0.83]	0.35
rs11957215*	*5p13.1*	G	0.28	7.08×10^−6^	0.72 [0.63–0.83]	0.35
rs7720838	*5p13.1*	C	0.37	2.19×10^−7^	0.71 [0.62–0.81]	0.45
rs10440635&	*5p13.1*	G	0.36	8.60×10^−8^	0.70 [0.61–0.80]	0.45

Note: Minor allele frequencies (MAF), allelic test p-values, and odds ratios (OR, shown for the minor allele) with 95% confidence intervals (CI) are shown.

* surrogate markers for rs4495224;

& surrogate marker for, rs7720838.

In the German replication cohort, the SNP rs10941508, which was in nearly complete linkage disequilibrium within the data of the Human HapMap project and served as surrogate marker for rs4495224, was also strongly associated with CD susceptibility. The frequencies of the minor G allele of the SNP rs10941508 were 0.30 in the CD population and 0.34 in the controls (p = 8.60×10^−8^; 0.70 [0.61–0.80]) (Table 4).

**Table 4:** Allele frequencies of the SNPs within the *5p13.1* in German replication cohort with Crohn's disease patients and controls.

Gene marker	Gene/region	Minor allele	Crohn's disease			Controls
			n = 1098			n = 1048
			MAF	p value	OR [95% CI]	MAF
rs10941508§	*5p13.1*	G	0.30	1.96×10^−3^	0.82 [0.72–0.93]	0.34

Note: Minor allele frequencies (MAF), allelic test p-values, and odds ratios (OR, shown for the minor allele) with 95% confidence intervals (CI) are shown.

§ surrogate marker for rs4495224.

### Genotype-phenotype analyses

So far, the phenotypic consequences of gene variants in the *5p13.1* region are unknown. We therefore performed a detailed genotype-phenotype correlation in the German IBD discovery cohort for which detailed phenotype data were available. In CD patients, the analysis revealed no significant associations of the SNPs rs4495224 and rs7720838 with phenotypic characteristics such as age, male-to-female-ratio, body mass index (BMI), family history, incidence of stenoses and fistulas, use of immunosuppressive agents, or extraintestinal manifestations (Table S6 and S7). Weak associations with disease onset <16 years in CD patients heterozygous for SNP rs4495224 (p = 0.036) and with less colonic involvement according to the Montreal classification [31] in heterozygous carriers of the rs7720838 variant compared to the wildtype patients (p = 0.023) did not fulfill the significance criteria after Bonferroni correction (Table S6 and S7). Similarly, in UC, no significant associations between these SNPs and the main disease characteristics were found after Bonferroni correction (Table S8 and S9).

### Analysis for epistasis between the 5p13.1 region and other CD susceptibility genes

Next, potential epistasis between the SNPs in the *5p13.1* region and other, replicated CD-associated gene variants was analyzed. This analysis included the three common *NOD2* variants p.Arg702Trp, p.Gly908Arg, and p.Leu1007fsX1008, 10 recently identified CD-associated *IL23R* variants [7], [8] 9 variants in *ATG16L1* [9], [11] and *SLC22A4/5* gene variants [32]. After Bonferroni correction, no evidence for epistasis between SNPs in the *5p13.1* region and gene variants in *NOD2*, *IL23R*, or *SLC22A4/5* was found (data not shown). In contrast, marked epistasis between the two SNPs of the *5p13.1* region (rs4495224 and rs7720838) and SNPs within the *ATG16L1* gene was demonstrated in the German CD discovery cohort (Table 5). The interactions were particularly strong between rs7720838 and *ATG16L1* polymorphisms, with p values ranging from 7.81×10^−3^ to 1.09×10^−7^ (Table 5). Strong interactions of rs7720838 occurred with rs13412102 in the 5′-flanking region (p = 1.09×10^−7^), with rs6431660 in the 5′-region of the gene (p = 2.29×10^−7^) and with rs2241880 (p.Thr300Ala) in the central region of *ATG16L1* (p = 5.99×10^−7^; Table 5). In the German CD discovery cohort, the epistasis between *ATG16L1* and rs4495224 was also strong, but less pronounced than that of rs7720838; the strongest interaction with rs4495224 involved rs6431660 (p = 8.37×10^−5^) and the coding SNP rs2241880 (p.Thr300Ala) (p = 3.81×10^−4^; Table 5). In addition, the *ATG16L1* SNP rs2241879, which was associated with CD in several studies [9]–[11], displayed strong interactions with both *5p13.1* SNPs (rs7720838: p = 1.10×10^−6^, rs4495224: p = 3.07×10^−4^).

**Table 5:** Epistasis analysis between SNPs rs4495224 and rs7720838 in the *5p13.1* region and SNPs within the *ATG16L1* gene regarding CD susceptibility in the German discovery study population.

	*5p13.1/PTGER4* SNPs	
*ATG16L1 SNPs*	rs4495224	rs7720838
	*P* value	*P* value
rs13412102	4.84×10^−4^	1.09×10^−7^
rs12471449	1.09×10^−2^ *	1.39×10^−2^ *
rs6431660	8.37×10^−5^	2.29×10^−7^
rs1441090*	8.36×10^−2^ *	5.66×10^−2^ *
rs2289472	3.05×10^−4^	1.34×10^−6^
rs2241880 (Thr300Ala)	3.81×10^−4^	5.99×10^−7^
rs2241879	3.07×10^−4^	1.10×10^−6^
rs3792106	2.65×10^−4^	1.40×10^−5^
rs4663396*	9.86×10^−3^ *	7.81×10^−3^ *

Note:

* All p values given are uncorrected for multiple comparisons. After applying Bonferroni correction, all associations remained significant (p<0.05) with the exceptions of those marked with an asterisk.

Despite the pronounced epistasis between *ATG16L1* and the *5p13.1* region regarding CD susceptibility in the German discovery cohort, no significant epistatic effect of these genetic regions on the CD or UC phenotype could be detected after Bonferroni correction (Table S8 and S9) which was partly related to the great number of interactions tested for (n = 189 for CD; n = 144 for UC).

To analyze if the epistasis between the *5p13.1* region and *ATG16L1* could be replicated in other CD populations, we investigated a U.S. American and a German CD replication cohort. In the U.S. American cohort, both *5p13.1* (Table 3) and *ATG16L1* (Table S10) were strongly associated with CD susceptibility. Using different genotyping platforms (Human Omni1-Quad chip from Illumina in the U.S. American and SNPlex technology from Applied Biosystems in the German replication cohort), a slightly different panel of SNPs in the *5p13.1* region and *ATG16L1* SNPs was available (Tables 3 and 4, Table S11). In the U.S. American study population, rs4532399 in the *5p13.1* region served as surrogate marker for rs4495224, and rs2289474 was used as surrogate marker for rs6431660. In the German replication cohort, rs10941508 in the *5p13.1* region served as surrogate marker for rs4495224. However, as shown in Tables S12 and S13, there was no significant epistasis detected between these gene markers, suggesting that the strong epistasis between *5p13.1* and *ATG16L1* found in the German CD discovery cohort is not a general phenomenon in Caucasian CD populations.

### Analysis of potential transcription factor binding sites in the 5p13.1 region harboring SNPs rs4495224 and rs7720838

The study of Libioulle et al. analyzed the influence of 26 SNPs within the gene desert on chromosome *5p13.1* regarding *PTGER4* gene expression [21]. They found that, amongst all analyzed SNPs, the CD risk alleles in rs4495224 and rs7720838 were most strongly associated with increased PTGER4 expression [21]. However, the underlying mechanisms explaining of how these SNPs might influence PTGER expression, were not examined so far. We therefore analyzed *in silico* for potential transcription factor binding sites in the genomic sequences containing SNPs rs4495224 or rs7720838 and the respective surrounding nucleotides. As depicted in table 6, several predicted transcription factor binding sites with high binding scores could be identified for the CD risk alleles in rs4495224 and rs7720838, suggesting a stronger transcription factor binding and hence higher expression of the neighboring *PTGER4* gene as it has been described by Libioulle et al. [21] for the respective CD risk alleles.

**Table 6:** Analysis of transcription factor binding sites in the DNA sequences surrounding SNPs rs4495224 and rs7720838 applying the program TFsearch.

rs4495224 **(approx. 200 kb upstream of *PTGER4*):**					
**5′-CAGAGTTTAAATTGG** *[A/C]* **ACTTCCCCTGAGGAC-3′** **plus strand**					
**3′-GTCTCAAATTTAACC** *[T/G]* **TGAAGGGGACTCCAG-5′** **minus strand**					
Transcription factor	Consensus sequence #(5′→3′)	DNA strand	Position relative to SNP (5′→3′)	Binding score risk allele‡	Binding score protective allele‡
**NF-κB** **(p50/p65 heterodimer)**	GGGAMTT**Y**CC	minus	−7 to +2	**92.2**	**86.0**
**c-Rel**	SGGRNWT**T**CC	minus	−7 to +2	**89.3**	**82.6**
**NF-κB** **p65 (RelA)**	GGGRATT**T**CC	minus	−7 to +2	***88.7***	***78.8***
**NF-κB2 (p52)**	NGGGACTT**T**CCA	minus	−8 to +3	**86.3**	**79.1**
MZF1	NGNGGGGA	plus	+4 to +11	82.6	82.6
**Elk-1**	NNNACMGGAAGT**N**CNN	minus	−12 to +3	80.5	80.5
**STATx**	TTCCCRKAA	plus	+3 to +11	79.8	79.8
**SRY**	AAACWAM	minus	+8 to +14	77.3	77.3
**Tst-1**	NNKGAWT**W**ANANTKN	minus	−7 to +7	**77.1**	**68.8**
**HSF2**	NG**A**ANNWTCK	plus	−2 to +7	**76.3**	**67.3**
**HSF1**	RG**A**ANTRRCN	plus	−2 to +7	***75.7***	***65.2***
**p300**	NNNRGGAGT**N**NNNS	minus	−9 to +4	73.3	77.2

rs7720838 **(approx. 193 kb upstream of *PTGER4)*:**					
**5′-CAGGGCTTTGACATG** *[T/G]* **CATCACCAATGCATC-3′** **plus strand**					
**3′-GTCCCGAAACTGTAC** *[A/C]* **GTAGTGGTTACGTAG-5′** **minus strand**					
Transcription factor	Consensus sequence # (5′→3′)	DNA strand	Position relative to SNP (5′→3′)	Binding score risk allele‡	Binding score protective allele‡
**GATA-1**	SNNGATN**N**NN	minus	−7 to +2	85.3	88.6
**XBP-1**	NNGNTG**A**CGTGKNNNWT	minus	−6 to +10	***83.2***	***73.3***
**GATA-2**	NNNGATR**N**NN	minus	−7 to +2	80.6	83.0
**Oct-1**	CWNAWTK**W**SATRYN	minus	−7 to +6	**79.6**	**73.5**
**CREB**	NNGNTGACG**Y**NN	minus	−9 to +2	79.5	82.8
**C/EBPb**	RNRTKNNG**M**AAKNN	minus	−8 to +5	78.6	79.2
**C/EBP**	NNTKTGGWNANN**N**	minus	−12 to 0	78.5	80.8
**MZF1**	NGNGGGGA	minus	−10 to −3	77.4	77.4
**c-Myc/Max**	NANC**A** CGTGNNW	minus	−4 to +7	***77.3***	***64.1***

Note:

# Nucleotides in the genomic sequences according with the consensus sequences are underlined and the polymorphic nucleotide is marked in **bold**.

‡ Predicted binding scores differing more than 5 points between CD risk alleles and protective alleles are depicted in **bold**. Scores differing 10 points and more are depicted in ***bold italic***. Binding score threshold for each allele was set to 75.0. Nucleotide codes: K = G or T, M = A or C, R = A or G, S = C or G, W = A or T, Y = C or T, N = A, G, C or T.

Interestingly, rs4495224 is part of a nearly perfect NF-κB consensus sequence (with only one nucleotide not matching the consensus sequence; Table 6). Accordingly, the highest binding scores for the DNA sequence containing the CD risk allele were predicted for the transcription factor NF-κB (p50/p65 heterodimer) as well as for the NF-κB subunits NF-κB p65 (RelA), NF-κB2 (p52) and c-Rel (Table 6). Binding of these factors to DNA containing the protective allele was predicted to be considerably weaker suggesting lower transcriptional activation of neighboring genes.

For rs7720838, the IBD-associated transcription factor XBP1, that has recently been identified as important modulator of intestinal inflammation [34], was predicted to bind strongly to a DNA sequence with the CD risk allele while predicted binding to a sequence with the protective allele was substantially lower (Table 6).

## Discussion

In summary, our study confirms the *5p13.1* region as susceptibility locus in CD. This finding is in agreement with the genome-wide association studies by Libioulle and co-workers [21] and Franke et al. [35]. In contrast, we could not replicate a contribution of the *5p13.1* region to UC susceptibility which was demonstrated by recent meta-analyses of GWAS [29], [36] and may be related to the limited sample size of our cohort and the weaker effect of the *5p13.1* region on UC susceptibility compared to CD susceptibility. Very recently, the largest meta-analysis ever performed in IBD, including more than 75,000 cases and controls, demonstrated an association with UC, which convincingly confirms that there is a very strong association of the *PTGER4* locus with CD (p = 1.81×10^−82^), while there is only a weak association with UC (p = 1.68×10^−5^ for the immunochip UC cohort) which reached only in all UC cohorts combined genome-wide significance (p = 1.36×10^−10^ for all UC cohorts combined) [37]. This clearly illustrates that extremely large cohorts are required to show significant results for weak associations as for *PTGER4* and UC. Following Bonferroni correction, we could not identify a specific IBD subphenotype associated with the investigated SNPs in the *5p13.1* region.

In CD, the observed ORs for the minor alleles of the analyzed *PTGER4* SNPs are below a value of 1.0 and thus, are most likely protective while the major alleles represent the CD risk alleles for both SNPs. The rs4495224 A and rs7720838 T risk alleles ( = major alleles) were associated with increased PTGER4 expression in the study of Libioulle et al. [21]. Although protective functions of EP4 against inflammation have been described, [22]–[24], [38], other studies reported a proinflammatory role for EP4 in models of rheumatoid arthritis or experimental autoimmune encephalitis [39]–[41]. Interestingly, EP4 has been shown to drive the differentiation of Th1 cells and proliferation of Th17 [40]–[42]. Since these two proinflammatory T cell subsets play very important roles in the pathogenesis of CD [43], increased expression of PTGER4 and therefore increased EP4 signaling in carriers of the CD risk alleles of the two SNPs rs4495224 and rs7720838 is plausible. The transcription factors NF-κB and XBP1 were identified as very likely candidates for binding to the respective genomic regions and thereby increasing PTGER4 expression. NF-κB is a transcription factor involved in many inflammatory signaling pathways and has been implicated in the pathogenesis of IBD [44]. XBP1 has very recently been described as an important transcription factor that links endoplasmatic reticulum stress to the development of intestinal inflammation [34]. However, further functional studies are necessary to clarify the influence of these transcription factors on *PTGER4* expression and to further elucidate the role of this chromosomal region in the CD pathogenesis.

Prostaglandins are arachidonic acid metabolites produced by the action of the enzymes cyclooxygenase-1 and -2 (COX-1 and COX-2) which have been identified to play a crucial role in the physiological regulation of inflammation and gastrointestinal homeostasis [45]–[47] as well as in the defense of the intestinal mucosa [48]. Moreover, a haplotype of prostaglandin synthase 2/cyclooxygenase 2 has been found to be involved in IBD susceptibility [25] and microsomal prostaglandin E synthase-1 is overexpressed in IBD [26]. Interestingly, prostaglandin 15-deoxy-delta(12,14)-PGJ2 attenuates the development of intestinal injury caused by dinitrobenzene sulphonic acid (DNBS) in rats [27].

Recently, *PTGER4* polymorphisms have been found to be associated with asthma [49] including aspirin-intolerant asthma [50], suggesting a role also in inflammation of the respiratory tract. Prostaglandin E2-EP4 signaling was further found to play a key role in skin immune responses by promoting migration and maturation of Langerhans cells, specialized antigen-presenting cells (APCs) [51]. Since APCs such as dendritic cells (DCs) are critical for the defense against intestinal bacterial microbiota [52], prostaglandin E2-EP4 signaling might also contribute to IBD via the regulation of intestinal DCs. Interestingly, we and others demonstrated that the capacity of lamina propria DCs to form transepithelial dendrites for sampling of luminal antigens depends on the chemokine receptor CX3CR1 [52] which was identified by us as an important genetic modifier of the CD phenotype [13].

In addition, we report strong epistasis between PTGER4 expression-modulating SNPs in the *5p13.1* region and the *ATG16L1* gene in the German discovery cohort. Based on the p-value of 1×10^−7^ for the strongest interaction (between rs7720838 and rs13412102), this is the strongest epistasis signal reported so far and nearly 3-log fold stronger than the most significant gene-gene interaction reported in the meta-analysis by Barrett et al. [53]. However, this gene-gene interaction was only observed in the German discovery population but not in the U.S. American and the German replication cohorts, suggesting that the epistasis between the two gene regions is not a general phenomenon contributing to CD susceptibility in all Caucasian populations. This is supported by the recent meta-analyses of GWAS [35], [53] which did not find epistasis between SNPs in the *PTGER4* and *ATG16L1* regions.

The lack of replication of the gene-gene interaction between *PTGER4* and *ATG16L1* regions may be related to population differences, although this is unlikely given the close genetic similarity between the South and North German population representing the discovery and the replication cohort, respectively. However, some minor genetic differences were shown between the populations analyzed in this study (e.g., association of CD with *DLG5* only in the North German population [54], [32] and association with *PHOX2B*, *NCF4* and *FAM92B* only in the North American population [10] but not the German population [55]). Furthermore, methodological issues could explain the lack of replication of the gene-gene interaction between *PTGER4* and *ATG16L1* since a different genotyping platform was used in the U.S. American and in the German CD replication cohorts requiring the use of surrogate markers instead of the original SNPs used in the German discovery population. However, given the high linkage disequilibrium between original SNPs and surrogate markers, this is very unlikely. The observed gene-gene interaction could also be coincidental which illustrates the need for extremely large sample sizes to find convincing association evidence and separate true signals from noise for complex trait loci that have small effect sizes.

The potential intergenic interaction between *ATG16L1* and the *5p13.1* region would be of particular interest since the exact functional consequences of polymorphisms in the *5p13.1* chromosomal region are largely unknown. In the study of Libioulle and co-workers [21], the disease-associated alleles were found to correlate with expression levels of *PTGER4* which was the gene located closest to the associated region. The finding of *PTGER4* as an important CD target gene in the *5p13.1* region is also in line with reports of *Ptger4* knock-out mice developing severe DSS-induced colitis [22], [23].

In summary, our study confirms the chromosome *5p13.1* region as a susceptibility locus in CD. For the first time, we demonstrate the strongly CD-associated *PTGER4* SNPs rs4495224 and rs7720838 as part of binding sites for NF-κB and XBP1, suggesting that these transcription factors may modulate PTGER4 expression. However, further functional assays are necessary to clarify if the SNPs analyzed in our study modulate binding of transcription factors and thereby regulating PTGER4 expression and IBD susceptibility. We could not identify a specific IBD phenotype associated with the SNPs rs4495224 and rs7720838, although the cohort used in this study convincingly demonstrated other strong association such as for the *NOD2* variant p.Leu1007fsX1008 with ileal CD involvement, stenosis and need for surgery [16], [17], [56], [57], suggesting that the sample size in CD was sufficient to detect clinically relevant associations. In addition, a strong epistasis signal between rs4495224 and rs7720838 with SNPs in the *ATG16L1* gene region was observed in the German CD discovery cohort. However, this gene-gene interaction could not be replicated in the North American CD cohort and in the German CD replication cohort, arguing against a major role of this interaction in the CD pathogenesis. Further functional studies are required to clarify the exact role of the *5p13.1* region in the CD pathogenesis.

## Supporting Information

Table S1 **Primer sequences, FRET probe sequences, and primer annealing temperatures used for genotyping of rs4495224 and rs7720838.**
(DOC)

Table S2 **Primer sequences used for sequence analysis of rs4495224 and rs7720838.**
(DOC)

Table S3 **Primer sequences and restriction enzymes used for genotyping of** ***NOD2*** **and** ***SLC22A4/5*** **variants.**
(DOC)

Table S4 **Primer sequences, FRET probe sequences, and primer annealing temperatures used for genotyping** ***IL23R*** **variants.**
(DOC)

Table S5 **Primer sequences, FRET probe sequences, and primer annealing temperatures used for genotyping** ***ATG16L1*** **variants.**
(DOC)

Table S6 **Association between rs4495224 genotype and CD disease characteristics based on the Montreal classification** [**31**] **.**
(DOC)

Table S7 **Association between rs7720838 genotype and CD disease characteristics based on the Montreal classification** [**31**] **.**
(DOC)

Table S8 **Association between rs4495224 genotype and UC disease characteristics based on the Montreal classification** [**31**] **.**
(DOC)

Table S9 **Association between rs7720838 genotype and UC disease characteristics based on the Montreal classification** [**31**] **.**
(DOC)

Table S10 **Allele frequencies of the SNPs within the** ***ATG16L1*** **gene in the North American replication cohort with Crohn's disease and controls.**
(DOC)

Table S11 **Allele frequencies of the SNPs within the** ***ATG16L1*** **gene region in the German replication cohort with Crohn's disease patients and controls.**
(DOC)

Table S12 **Epistasis analysis between SNPs rs4495224 and rs7720838 in the** ***5p13.1*** **region and SNPs within the** ***ATG16L1*** **gene regarding CD susceptibility in the North American (NIDDK IBD Genetics Consortium) replication cohort.**
(DOC)

Table S13 **Epistasis analysis between SNPs rs10941508 and rs7720838 in the** ***5p13.1*** **region and SNPs within the** ***ATG16L1*** **gene regarding CD susceptibility in the German replication cohort.**
(DOC)
