# Supplementary material for: PTGER4 Expression-Modulating Polymorphisms in the 5p13.1 Region Predispose to Crohn's Disease and Affect NF-κB and XBP1 Binding Sites
Source: PLoS One. 2012 Dec 27;7(12):e52873. doi: 10.1371/journal.pone.0052873 (PMC3531335; doi:10.1371/journal.pone.0052873)
Supplement: Table S1 — Primer sequences, FRET probe sequences, and primer annealing temperatures used for genotyping of rs4495224 and rs7720838. (DOC) [file pone.0052873.s001.doc]

**Supplementary Table S1.** Primer sequences, FRET probe sequences, and primer annealing temperatures used for genotyping of rs4495224 and rs7720838.

| **Polymorphism** | **Primer sequences** | **Primer annealing** | **FRET probe sequences** |
| --- | --- | --- | --- |
| rs4495224 | TGCTTTTTGTTGTTGTTTTTAAGGTA | 60 °C | CACAGAGTTTAAATTGGCACTT-FL |
|  | GGAGGTGAAGTCCTTGAACTTG |  | LC610-CCCCTGAGGACCTGGACTCCA |
| rs7720838 | GGAAAGACTCACCAAGGTTCAT | 60 °C | CATTGGTGATGCCATGTCAA-FL |
|  | TGAGGAGAGAAGAGTGAGAGGC |  | LC640-GCCCTGGATTAGGACTCAGAAGACCTCA |

Note: FL: Fluorescein; LC610: LightCycler Red 610; LC640: LightCycler Red 640. The polymorphic position within the sensor probe is underlined. A phosphate is linked to the 3'-end of the acceptor probe to prevent elongation by the DNA polymerase.
