# Supplementary material for: PTGER4 Expression-Modulating Polymorphisms in the 5p13.1 Region Predispose to Crohn's Disease and Affect NF-κB and XBP1 Binding Sites
Source: PLoS One. 2012 Dec 27;7(12):e52873. doi: 10.1371/journal.pone.0052873 (PMC3531335; doi:10.1371/journal.pone.0052873)
Supplement: Table S2 — Primer sequences used for sequence analysis of rs4495224 and rs7720838. (DOC) [file pone.0052873.s002.doc]

**Supplementary Table S2.** Primer sequences used for sequence analysis of rs4495224 and rs7720838.

| **Polymorphism** | **Primer sequences** |
| --- | --- |
| rs4495224 | TGTTATACAGAGCAGCAGAACTAGG |
|  | CTGGGAAGTACAGCCCTTTATTC |
| rs7720838 | GGGTGAATGATCAATGTAACCG |
|  | AGATCAGGGTGAGTCCAGAGAAT |
