# Supplementary material for: PTGER4 Expression-Modulating Polymorphisms in the 5p13.1 Region Predispose to Crohn's Disease and Affect NF-κB and XBP1 Binding Sites
Source: PLoS One. 2012 Dec 27;7(12):e52873. doi: 10.1371/journal.pone.0052873 (PMC3531335; doi:10.1371/journal.pone.0052873)
Supplement: Table S3 — Primer sequences and restriction enzymes used for genotyping of NOD2 and SLC22A4/5 variants. (DOC) [file pone.0052873.s003.doc]

**Supplementary Table S3. Primer sequences and restriction enzymes used for genotyping of *NOD2* and *SLC22A4/5* variants.**

| **Polymorphism** | **Primer sequences** | **Restriction enzyme** | **Length of fragments** |
| --- | --- | --- | --- |
| rs2066844 | TGGGGCCTGCTGGCTGAGTG | *Msp I* | C allele: 76 bp + 45 bp |
| (*NOD2* p.Arg702Trp) | GTGCAGCTGGCGGGATGGAG |  | T allele: 121 bp |
| rs2066845 | TCTGGCTGGGACTGCAGAGG | *BstU I* | G allele: 131 bp |
| (*NOD2* p.Gly908Arg) | CCCCTCGTCACCCACTCTGTCGC |  | C allele: 109 bp + 22 bp |
| rs2066847 | GGCTAACTCCTGCAGTCTCTTTAACTGG | *Mwo I* | non-insC allele: 168 bp |
| (*NOD2* p.Leu1007fsX1008) | ACTTCCAGGATGGTGTCATTCCGCTCAAGG |  | insC allele: 143 bp + 26 bp |
| [rs1050152](http://snpper.chip.org/bio/view-snp/1050152) | CGTCATGGGTAGTCTGACTGTCCTGATTGGGATC | *BamH I* | C allele: 30 bp + 88 bp |
| (*SLC22A4* 1672C→T) | tcctacttaccatttcactttctgcatctgctctaagg |  | T allele: 118 bp |
| [rs2631367](http://snpper.chip.org/bio/view-snp/2631367) | GCGCCGCTCTGCCTGCCAG | *Msp I* | G allele: 44 bp + 83 bp |
| (*SLC22A5* –207G→C) | agggtaggctcgcgagctgacacc |  | C allele: 127 bp |

Note: The underlined bases in the primers differ from the original sequences and served to introduce a restriction site or to disrupt a natural site within the primer sequence.
