# Supplementary material for: PTGER4 Expression-Modulating Polymorphisms in the 5p13.1 Region Predispose to Crohn's Disease and Affect NF-κB and XBP1 Binding Sites
Source: PLoS One. 2012 Dec 27;7(12):e52873. doi: 10.1371/journal.pone.0052873 (PMC3531335; doi:10.1371/journal.pone.0052873)
Supplement: Table S4 — Primer sequences, FRET probe sequences, and primer annealing temperatures used for genotyping IL23R variants. (DOC) [file pone.0052873.s004.doc]

**Supplementary Table S4. Primer sequences, FRET probe sequences, and primer annealing temperatures used for genotyping *IL23R* variants.**

| **Polymorphism** | **Primer sequences** | **Primer annealing** | **FRET probe sequences** |
| --- | --- | --- | --- |
| rs1004819 | TTCTAGGACCCTTTTGGC | 58°C | AGATAGCACAGTAAGAATCACAGC-FL |
|  | TTTGCAAAAATATGAACTCATTCAA |  | LC610-AAGCAGGTCAGTGCAATCCAGATT |
| rs7517847 | CTGCCAATTCCCTAAACA | 59°C | AAGGCCTCAGCTACACCTGTAT-FL |
|  | GACAGCCCATAAAGATACAAACA |  | LC670-GCTAGAAACTGGAGCCCTCCCAACTC |
| rs10489629 | AGGTGTCATTACCCACCAGCA | 58°C | GTCAGCCACATTTGGTAGTG-FL |
|  | CTGCCTAGCAAGATTATGCAA |  | LC640-TTAGCGCTACTTTACAAAAGCGGAAAC |
| rs2201841 | AGGAATGTAGGCAGCCTCTAG | 59°C | TATAGAAGATGATGACCTCAAGAAA-FL |
|  | GTGCTGGGCTTACAGGCAG |  | LC640-GCATAATCATAGGCCAGGCATGGTG |
| rs11465804 | CACATGGAATTCTGGGCTA | 57°C | GTAGTCTTTTAGTAATTGCC-FL |
|  | AAGGCATATCTTATTGTCCAGAAA |  | LC610-ACCCATCATACTGAAAAAATCACATCAGG |
| rs11209026 | CTTTCCTTTCATTAGACAACAGAGG | 56°C | ACAGATCATTCCAAACTGGGT-FL |
|  | AACTGAAATGACTAAATTTTGGTGA |  | LC640-GTTTTTGCAGAATTTCTGTTTTCTGATTT |
| rs1343151 | CGAAAGAAAGATTATTTCATGAAGC | 57°C | TTGAATGTTCTTTGCCAAATTG-FL |
|  | AGTGTGTCCAGTTGCTCAC |  | LC670-CACTGAGTTTCTCCTTCTTGCCATCA |
| rs10889677 | TCCATGCCTTTTTAATTTTAGC | 59°C | TTCTGCCTCATTTCTTAAAATTAGA-FL |
|  | GACTCTATAAAAAATACATGAGGCGTC |  | LC610-TAAGGTCCCGAAGGTGGAACATGC |
| rs11209032 | GGTGTTTGTTTTATCTTGTACGC | 59°C | CTTTGCAATGGCAGATGGAAG-FL |
|  | CAATCCGGTGGTTCTTCACAG |  | LC640-TGGCAATAAATGCAATTCAGCTTGAAG |
| rs1495965 | CAGGAATAAATGTGAATGAGAACAGAC | 58°C | GAAAATGTTCTCTTCCTCCACAG-FL |
|  | CAATATTTATATCTCCCATGGCTC |  | LC670-GGATTAACATTTGCTTCTGGCAGC |

Note: FL: Fluorescein, LC610: LightCycler-Red 610; LC640: LightCycler-Red 640; LC670: LightCycler-Red 670. The polymorphic position within the sensor probe is underlined. A phosphate is linked to the 3'-end of the anchor probe to prevent elongation by the DNA polymerase in the PCR.
