# Supplementary material for: PTGER4 Expression-Modulating Polymorphisms in the 5p13.1 Region Predispose to Crohn's Disease and Affect NF-κB and XBP1 Binding Sites
Source: PLoS One. 2012 Dec 27;7(12):e52873. doi: 10.1371/journal.pone.0052873 (PMC3531335; doi:10.1371/journal.pone.0052873)
Supplement: Table S5 — Primer sequences, FRET probe sequences, and primer annealing temperatures used for genotyping ATG16L1 variants. (DOC) [file pone.0052873.s005.doc]

**Supplementary Table S5. Primer sequences, FRET probe sequences, and primer annealing temperatures used for genotyping *ATG16L1* variants.**

| **Polymorphism** | **Primer sequences** | **Primer annealing** | **FRET probe sequences** |
| --- | --- | --- | --- |
| rs13412102 | CAGCTCCTTTCATTCCCAC | 60 °C | LC640-ACCCTGCCCTAGAACGTAC |
|  | CTTCCCTCTCTCCTTCACC |  | GACACTTCCTTCAATAAATCTCTCATGTCT-FL |
| rs12471449 | GCTGGGTTGGAATCTGC | 60 °C | CATACCTATAAAGCACTTACTGTG-FL |
|  | TAGCACTTATCACCTCTGATATCTTAG |  | LC610-GCAAGACACCGTTGTCAGTATTAATTC |
| rs6431660 | GTATTTTGGTTAATTTTAGGTGTAG | 57 °C | CAGATAGAAATGTTTTCAGTTTTC-FL |
|  | GCTGCATATTCCAAGCAC |  | LC670-GGATGAATTTAGACGAAAATGGTGAAAC |
| rs1441090 | GAGGGAAGAAGGAACCATAGAT | 60 °C | LC640-GAGACCCCCAACCCTTCA |
|  | CTTGGGCATTCACACAGGT |  | GGAGGCAGCTCTTGCTCATTAAAGCTGGT-FL |
| rs2289472 | GAATATGTTTCCCACTTAGTTCC | 57 °C | LC610-GTTGGGTTTCTCTTGGCT |
|  | ATTGTGAGGTTAGTTGTCTAATGC |  | CCCACTGTTTTCTGGCAGTTTGGTACTG-FL |
| rs2241880 | ATTTGTCTTTATGTTATTTCTTAGGAGACG | 60 °C | LC610-CCAGGATGAGCATCCACATTGT |
|  | GTAACAAATTTTGTCCTCTGAAACTA |  | GTAGCTGGTACCCTCACTTCTTTACCAGA-FL |
| rs2241879 | TTTGCCCCATCCCTCAT | 60 °C | CCAGGCTCTGTCACCATATCA -FL |
|  | GCCTTGTGTGTCTTCGTAAGTAT |  | LC670-GCGTGGTAGGGTTCGGGGCT |
| rs3792106 | GTTCATCTTCAATGTGTCGAGC | 60 °C | TGGCTTTACAAAAGTCTAAACC-FL |
|  | CTTTTACTAAGCTGAGAGCTGACTAC |  | LC640-CAATACAACATACCCTCATTTTCCAACC |
| rs4663396 | AACAGAGGAAGTAATGTTAAGATGA | 60 °C | LC670-GATCACACTACTATACCCCACAC |
|  | GCAGATCCCTTGAGGTCA |  | GAAGGCGGAGGTTGCAGTGAGCTG-FL |

Note: FL: Fluorescein, LC610: LightCycler-Red 610; LC640: LightCycler-Red 640; LC670: LightCycler-Red 670. The polymorphic position within the sensor probe is underlined. A phosphate is linked to the 3'-end of the anchor probe to prevent elongation by the DNA polymerase in the PCR
