# Supplementary material for: PTGER4 Expression-Modulating Polymorphisms in the 5p13.1 Region Predispose to Crohn's Disease and Affect NF-κB and XBP1 Binding Sites
Source: PLoS One. 2012 Dec 27;7(12):e52873. doi: 10.1371/journal.pone.0052873 (PMC3531335; doi:10.1371/journal.pone.0052873)
Supplement: Table S6 — Association between rs4495224 genotype and CD disease characteristics based on the Montreal classification [31] . (DOC) [file pone.0052873.s006.doc]

**Supplementary Table S6.** Association between rs4495224 genotype and CD disease characteristics based on the Montreal classification ^31^.

| **rs4495224 genotype** | (1) | (2) | (3) | **(1) vs. (2)** | **(1) vs. (3)** | **(1) vs. (2) + (3)** |
| --- | --- | --- | --- | --- | --- | --- |
| **status** | **AA** | **AC** | **CC** | **p value** | **p value** | **p value** |
|  | n=433 | n=350 | n=59 | **OR (95% CI)** | **OR (95% CI)** | **OR (95% CI)** |
| **Male sex** | 187/364 (51.4%) | 155/300 (51.7%) | 27/52 (51.9%) | 1.000 | 1.000 | 0.940 |
|  |  |  |  | 1.01 (0.74-1.37) | 1.02 (0.57-1.83) | 1.01 (0.76-1.36) |
| **Age at diagnosis (yrs)** |  |  |  |  |  |  |
| Mean ± SD | 27.4±11.0 | 28.0±12.5 | 27.0±11.8 | 0.545 | 0.862 | 0.620 |
| Range | 9-70 | 1-78 | 7-71 |  |  |  |
| **Disease duration (yrs)** |  |  |  |  |  |  |
| Mean ± SD | 12.2±8.5 | 11.3±8.4 | 10.6±9.2 | 0.279 | 0.376 | 0.217 |
| Range | 0-40 | 1-33 | 1-44 |  |  |  |
| **Age at diagnosis** |  |  |  |  |  |  |
| ≤16 years (A1) | 113/317 (35.6%) | 66/244(27.0%) | 18/41 (43.9%) | **0.036** | 0.306 | 0.118 |
|  |  |  |  | 0.67 (0.47-0.96) | 1.41 (0.73-2.73) | 0.75 (0.53-1.06) |
| 17-40 years (A2) | 177/317 (55.8%) | 152/244 (62.3%) | 19/41 (46.3%) | 0.142 | 0.317 | 0.322 |
|  |  |  |  | 1.31 (0.93-1.84) | 0.68 (0.36-1.31) | 1.19 (0.86-1.64) |
| > 40 years (A3) | 27/317 (8.5%) | 26/244 (10.7%) | 4/41 (9.8%) | 0.467 | 0.768 | 0.407 |
|  |  |  |  | 1.28 (0.73-2.26) | 1.16 (0.38-3.50) | 1.26 (0.73-2.18) |
| **Location** |  |  |  |  |  |  |
| Terminal ileum (L1) | 43/318 (13.5%) | 44/254 (17.3%) | 5/45 (11.1%) | 0.241 | 0.816 | 0.366 |
|  |  |  |  | 1.34 (0.85-2.12) | 0.80 (0.30-2.14) | 1.25 (0.80-1.95) |
| Colon (L2) | 45/318 (14.2%) | 34/254 (13.4%) | 8/45 (17.8%) | 0.809 | 0.502 | 1.000 |
|  |  |  |  | 0.94 (0.58-1.51) | 1.31 (0.57-3.00) | 0.99 (0.63-1.56) |
| Ileocolon (L3) | 226/318 (71.1%) | 170/254 (66.9%) | 30/45 (66.7%) | 0.316 | 0.601 | 0.296 |
|  |  |  |  | 0.82 (0.58-1.18) | 0.81 (0.42-1.58) | 0.82 (0.58-1.16) |
| Upper GI (L4) | 4/318 (1.3%) | 6/254 (2.4%) | 2/45 (4.4%) | 0.351 | 0.163 | 0.250 |
|  |  |  |  | 1.90 (0.53-6.80) | 3.65 (0.65-20.53) | 2.16 (0.64-7.24) |
| Ileocolonic | 269/318 (84.6%) | 214/254 (84.3%) | 35/45 (77.8%) | 0.908 | 0.279 | 0.662 |
| Involvement |  |  |  | 0.97 (0.62-1.53) | 0.64 (0.30-1.37) | 0.91 (0.59-1.39) |
| **Behaviour** ^1^ |  |  |  |  |  |  |
| Non-stricturing, | 58/312 (18.6%) | 60/243 (24.7%) | 10/41 (24.4%) | 0.094 | 0.400 | 0.073 |
| Non-penetrat. (B1) |  |  |  | 1.44 (0.95-2.16) | 1.41 (0.66-3.04) | 1.43 (0.97-2.12) |
| Stricturing (B2) | 89/312 (28.5%) | 56/243 (23.0%) | 14/41 (34.1%) | 0.173 | 0.468 | 0.308 |
|  |  |  |  | 0.75 (0.51-1.10) | 1.30 (0.65-2.59) | 0.82 (0.57-1.18) |
| Penetrating (B3) | 165/312 (52.9%) | 127/243 (52.2%) | 17/41 (41.5%) | 0.932 | 0.186 | 0.623 |
|  |  |  |  | 0.96 (0.70-1.36) | 0.63 (0.33-1.22) | 0.92 (0.66-1.26) |
| **Use of immuno-** | 180/222 (81.1%) | 148/181 (81.8%) | 20/27 (74.1%) | 0.898 | 0.441 | 1.000 |
| **suppressive agents** ^2^ |  |  |  | 1.05 (0.63-1.73) | 0.67 (0.26-1.68) | 0.98 (0.61-1.59) |
| **Surgery because of** | 163/300 (54.3%) | 136/237 (57.4%) | 20/40 (50.0%) | 0.486 | 0.617 | 0.675 |
| **CD** ^3^ |  |  |  | 1.13 (0.80-1.59) | 0.84 (0.43-1.63) | 1.08 (0.78-1.50) |
| **Fistulas** | 165/307 (53.7%) | 127/238 (53.4%) | 17/41 (41.5%) | 0.931 | 0.183 | 0.620 |
|  |  |  |  | 0.98 (0.70-1.38) | 0.61 (0.31-1.18) | 0.92 (0.66-1.27) |
| **Stenosis** | 199/306 (65.0%) | 151/243 (62.1%) | 26/41 (63.4%) | 0.532 | 0.863 | 0.549 |
|  |  |  |  | 0.88 (0.62-1.25) | 0.93 (0.47-1.83) | 0.89 (0.64-1.24) |

Note: For each variable, the number of patients with detailed information on this particular disease variable and included in the analysis is given.

^1^ Disease behaviour was defined according to the Montreal classification ^31^. A stricturing disease phenotype was defined as presence of stenosis without penetrating disease. The diagnosis of stenosis was made surgically, endoscopically, or radiologically (using MRI enteroclysis).

^2^ Immunosuppressive agents included azathioprine, 6-mercaptopurine, methotrexate, infliximab and/or adalimumab.

^3^ Only surgery related to CD-specific problems (e.g. fistulectomy, colectomy, ileostomy) was included.
