# Supplementary material for: PTGER4 Expression-Modulating Polymorphisms in the 5p13.1 Region Predispose to Crohn's Disease and Affect NF-κB and XBP1 Binding Sites
Source: PLoS One. 2012 Dec 27;7(12):e52873. doi: 10.1371/journal.pone.0052873 (PMC3531335; doi:10.1371/journal.pone.0052873)
Supplement: Table S7 — Association between rs7720838 genotype and CD disease characteristics based on the Montreal classification [31] . (DOC) [file pone.0052873.s007.doc]

**Supplementary Table S7.** Association between rs7720838 genotype and CD disease characteristics based on the Montreal classification ^31^.

| **rs7720838 genotype** | **(1)** | **(2)** | **(3)** | **(1) vs. (2)** | **(1) vs. (3)** | **(1) vs. (2) + (3)** |
| --- | --- | --- | --- | --- | --- | --- |
| **status** | **TT** | **GT** | **GG** | **p value** | **p value** | **p value** |
|  | n=315 | n=419 | n=104 | **OR (95% CI)** | **OR (95% CI)** | **OR (95% CI)** |
| Male sex | 141/271 (52.0%) | 173/349 (49.6%) | 51/92 (55.4%) | 0.571 | 0.629 | 0.758 |
|  |  |  |  | 0.91 (0.66-1.24) | 1.15 (0.71-1.84) | 0.95 (0.70-1.29) |
| **Age at diagnosis (yr)** |  |  |  |  |  |  |
| Mean ± SD | 27.6±11.2 | 27.8±12.6 | 27.0±9.4 | 0.884 | 0.630 | 0.992 |
| Range | 6-70 | 7-78 | 1-52 |  |  |  |
| **Disease duration (yr)** |  |  |  |  |  |  |
| Mean ± SD | 12.6±8.7 | 11.7±8.5 | 9.6±7.5 | 0.305 | **0.015** | 0.115 |
| Range | 1-37 | 0-44 | 1-40 |  |  |  |
| **Age at diagnosis** |  |  |  |  |  |  |
| ≤16 years (A1) | 87/236 (36.9%) | 85/289 (29.4%) | 25/75 (33.3%) | 0.076 | 0.679 | 0.092 |
|  |  |  |  | 0.71 (0.49-1.03) | 0.86 (0.49-1.48) | 0.74 (0.52-1.05) |
| 17-40 years (A2) | 129/236 (54.7%) | 172/289 (59.5%) | 45/75 (60.0%) | 0.287 | 0.427 | 0.237 |
|  |  |  |  | 1.22 (0.86-1.73) | 1.24 (0.73-2.11) | 1.22 (0.88-1.70) |
| > 40 years (A3) | 20/236 (8.5%) | 32/289 (11.1%) | 5/75 (6.7%) | 0.379 | 0.808 | 0.569 |
|  |  |  |  | 1.34 (0.75-2.42) | 0.77 (0.28-2.13) | 1.22 (0.69-2.16) |
| **Location** |  |  |  |  |  |  |
| Terminal ileum (L1) | 31/238 (13.0%) | 54/299 (18.1%) | 7/77 (9.1%) | 0.123 | 0.326 | 0.298 |
|  |  |  |  | 1.47 (0.91-2.38) | 0.61 (0.26-1.44) | 1.29 (0.81-2.06) |
| Colon (L2) | 42/238 (17.6%) | 32/299 (10.7%) | 13/77 (16.9%) | **0.023** | 1.000 | 0.057 |
|  |  |  |  | 0.56 (0.34-0.92) | 0.95 (0.48-1.88) | 0.63 (0.40-1.00) |
| Ileocolon (L3) | 162/238 (68.1%) | 206/299 (68.9%) | 55/77 (71.4%) | 0.852 | 0.671 | 0.722 |
|  |  |  |  | 1.04 (0.72-1.50) | 1.17 (0.67-2.06) | 1.06 (0.75-1.51) |
| Upper GI (L4) | 3/238 (1.3%) | 7/299 (2.3%) | 2/77 (2.6%) | 0.524 | 0.599 | 0.385 |
|  |  |  |  | 1.88 (0.48-7.34) | 2.10 (0.34-12.74) | 1.92 (0.51-7.17) |
| Ileocolonic | 193/238 (81.1%) | 260/299 (87.0%) | 62/77 (80.5%) | 0.073 | 1.000 | 0.144 |
| Involvement |  |  |  | 1.55 (0.97-2.48) | 0.96 (0.50-1.85) | 1.39 (0.90-2.15) |
| **Behaviour** ^1^ |  |  |  |  |  |  |
| Non-stricturing, | 45/230 (19.6%) | 65/291 (22.3%) | 18/72 (25.0%) | 0.452 | 0.323 | 0.358 |
| Non-penetrat. (B1) |  |  |  | 1.18 (0.77-1.81) | 1.37 (0.73-2.56) | 1.22 (0.81-1.83) |
| Stricturing (B2) | 62/230 (27.0%) | 77/291 (26.5%) | 21/72 (29.2%) | 0.921 | 0.763 | 1.000 |
|  |  |  |  | 0.97 (0.66-1.44) | 1.12 (0.62-2.00) | 1.00 (0.69-1.45) |
| Penetrating (B3) | 123/230 (53.4%) | 149/291 (51.2%) | 33/72 (45.8%) | 0.659 | 0.281 | 0.449 |
|  |  |  |  | 0.91 (0.65-1.29) | 0.74 (0.43-1.25) | 0.87 (0.63-1.22) |
| **Use of immuno-** | 129/160 (80.6%) | 175/214 (81.8%) | 41/53 (77.4%) | 0.790 | 0.693 | 1.000 |
| **suppressive agents** ^2^ |  |  |  | 1.08 (0.64-1.82) | 0.82 (0.39-1.74) | 1.02 (0.62-1.67) |
| **Surgery because of** | 117/223 (52.5%) | 162/279 (58.1%) | 37/72 (51.4%) | 0.240 | 0.893 | 0.344 |
| **CD** ^3^ |  |  |  | 1.25 (0.88-1.79) | 0.96 (0.56-1.63) | 1.19 (0.85-1.66) |
| **Fistulas** | 123/226 (54.4%) | 149/285 (52.3%) | 33/72 (45.8%) | 0.656 | 0.224 | 0.444 |
|  |  |  |  | 0.92 (0.65-1.30) | 0.71 (0.42-1.21) | 0.87 (0.62-1.22) |
| **Stenosis** | 140/224 (62.5%) | 189/290 (65.2%) | 45/74 (60.8%) | 0.578 | 0.890 | 0.660 |
|  |  |  |  | 1.12 (0.78-1.61) | 0.93 (0.54-1.60) | 1.08 (0.76-1.52) |

Note: For each variable, the number of patients included is given. Footnotes: see Supplemental Table S6 for details
