# Supplementary material for: PTGER4 Expression-Modulating Polymorphisms in the 5p13.1 Region Predispose to Crohn's Disease and Affect NF-κB and XBP1 Binding Sites
Source: PLoS One. 2012 Dec 27;7(12):e52873. doi: 10.1371/journal.pone.0052873 (PMC3531335; doi:10.1371/journal.pone.0052873)
Supplement: Table S8 — Association between rs4495224 genotype and UC disease characteristics based on the Montreal classification [31] . (DOC) [file pone.0052873.s008.doc]

**Supplementary Table S8. Association between rs4495224 genotype and UC disease characteristics based on the Montreal classification ^31^.**

| **rs4495224 genotype** | (1) | (2) | **(3)** | **(1) vs. (2)** | **(1) vs. (3)** | **(1) vs. (2) + (3)** |  |
| --- | --- | --- | --- | --- | --- | --- | --- |
| **status** | **AA** | **AC** | **CC** | **p value** | **p value** | **p value** |  |
|  | n=196 | n=209 | n=59 | **OR (95% CI)** | **OR (95% CI)** | **OR (95% CI)** |  |
| Male sex **n= (%)** | 52.0% | 53.1% | 47.7% | 0.909 | 0.732 | 1.000 |  |
|  |  |  |  | 1.04 (0.67-1.63) | 0.84 (0.43-1.65) | 1.00 (0.65-1.52) |  |
| **Body mass index** (kg/m^2^) |  |  |  |  |  |  |  |
| Mean ± SD | 23.7±4.1 | 24.0±4.5 | 24.2±3.7 | 0.742 | 0.600 | 0.653 |  |
| Range | 15-37 | 16-41 | 16-31 |  |  |  |  |
| **Age at diagnosis** (yrs) |  |  |  |  |  |  |  |
| Mean ± SD | 31.5±13.9 | 32.0±13.1 | 35.2±12.8 | 0.784 | 0.147 | 0.449 |  |
| Range | 9-81 | 11-68 | 15-64 |  |  |  |  |
| **Disease duration** (yrs) |  |  |  |  |  |  |  |
| Mean ± SD | 10.6±7.9 | 10.8±8.3 | 8.6±5.7 | 0.870 | 0.156 | 0.763 |  |
| Range | 1-40 | 1-35 | 2-28 |  |  |  |  |
| **Location** |  |  |  |  |  |  |  |
| Ulcerative proctitis (E1) | 13/123 (10.6%) | 16/131 (12.2%) | 6/40 (15.0%) | 0.695 | 0.399 | 0.586 |  |
|  |  |  |  | 1.23 (0.57-2.67) | 1.56 (0.55-4.42) | 1.31 (0.63-2.70) |  |
| Left-sided UC (E2) | 47/123 (38.2%) | 44/131 (33.6%) | 13/40 (32.5%) | 0.513 | 0.575 | 0.390 |  |
|  |  |  |  | 0.82 (0.49-1.37) | 0.78 (0.37-1.66) | 0.81 (0.50-1.31) |  |
| Extensive UC (E3) | 63/123 (51.2%) | 71/131 (54.2%) | 21/40 (52.5%) | 0.706 | 1.000 | 0.723 |  |
|  |  |  |  | 1.13 (0.69-1.84) | 1.05 (0.51-2.15) | 1.11 (0.70-1.76) |  |
| **Age at diagnosis** |  |  |  |  |  |  |  |
| A1 <17 years | 5/76 (6.6%) | 8/86 (9.3%) | 1/29 (3.4%) | 0.575 | 1.000 | 1.000 |  |
|  |  |  |  | 1.46 (0.45-4.66) | 0.51 (0.06-4.54) | 1.21 (0.39-3.75) |  |
| A2 17-40 years | 61/76 (80.3%) | 62/86 (72.1%) | 19/29 (65.5%) | 0.271 | 0.129 | 0.175 |  |
|  |  |  |  | 0.63 (0.30-1.33) | 0.47 (0.18-1.21) | 0.59 (0.29-1.17) |  |
| A3 >40 years | 10/76 (13.2%) | 16/86 (18.6%) | 9/29 (31.0%) | 0.396 | **0.047** | 0.181 |  |
|  |  |  |  | 1.51 (0.64-3.56) | 2.97 (1.06-8.32) | 1.83 (0.82-4.08) |  |
| **Use of immuno-** | 53/75 (70.7%) | 69/88 (78.4%) | 23/29 (79.3%) | 0.281 | 0.464 | 0.231) | |
| **suppressive agents**^1^ |  |  |  | 1.51 (0.74-3.07) | 1.59 (0.57-4.44) | 1.53 (0.78-2.97 | |

Note: ^1^ Immunosuppressive agents included azathioprine, 6-mercaptopurine, and/or infliximab.
