# Supplementary material for: PTGER4 Expression-Modulating Polymorphisms in the 5p13.1 Region Predispose to Crohn's Disease and Affect NF-κB and XBP1 Binding Sites
Source: PLoS One. 2012 Dec 27;7(12):e52873. doi: 10.1371/journal.pone.0052873 (PMC3531335; doi:10.1371/journal.pone.0052873)
Supplement: Table S9 — Association between rs7720838 genotype and UC disease characteristics based on the Montreal classification [31] . (DOC) [file pone.0052873.s009.doc]

**Supplementary Table S9. Association between rs7720838 genotype and UC disease characteristics based on the Montreal classification ^31^.**

| **rs7720838 genotype** | **(1)** | **(2)** | **(3)** | **(1) vs. (2)** | **(1) vs. (3)** | **(1) vs. (2) + (3)** |
| --- | --- | --- | --- | --- | --- | --- |
| **status** | **TT** | **GT** | **GG** | **p value** | **p value** | **p value** |
|  | n=141 | n=232 | n=91 | **OR (95% CI)** | **OR (95% CI)** | **OR (95% CI)** |
| Male sex **n= (%)** | 54.1% | 51.2% | 52.8% | 0.715 | 0.881 | 0.731 |
|  |  |  |  | 0.89 (0.55-1.44) | 0.95 (0.52-1.72) | 0.91 (0.58-1.42) |
| **Body mass index** (kg/m^2^) |  |  |  |  |  |  |
| Mean ± SD | 23.3±3.7 | 24.1±4.7 | 24.3±3.6 | 0.311 | 0.265 | 0.230 |
| Range | 15-36 | 16-41 | 16-31 |  |  |  |
| **Age at diagnosis** (yrs) |  |  |  |  |  |  |
| Mean ± SD | 32.6±13.5 | 31.4±13.7 | 33.0±12.9 | 0.549 | 0.856 | 0.697 |
| Range | 15-81 | 9-68 | 16-64 |  |  |  |
| **Disease duration** (yrs) |  |  |  |  |  |  |
| Mean ± SD | 9.4±6.7 | 11.8±8.9 | 9.0±6.1 | 0.066 | 0.725 | 0.186 |
| Range | 1-30 | 1-40 | 2-28 |  |  |  |
| **Location** |  |  |  |  |  |  |
| Ulcerative proctitis (E1) | 6/90 (6.7%) | 19/140 (13.6%) | 10/60 (16.7%) | 0.129 | 0.062 | 0.078 |
|  |  |  |  | 2.20 (0.84-5.74) | 2.80 (0.96-8.17) | 2.37 (0.95-5.94) |
| Left-sided UC (E2) | 34/90 (37.8%) | 47/140 (33.6%) | 23/60 (38.3%) | 0.572 | 1.000 | 0.692 |
|  |  |  |  | 0.83 (0.48-1.45) | 1.02 (0.52-2.01) | 0.89 (0.53-1.48) |
| Extensive UC (E3) | 50/90 (55.6%) | 74/140 (52.8%) | 27/60 (45.0%) | 0.786 | 0.244 | 0.448 |
|  |  |  |  | 0.90 (0.53-1.53) | 0.65 (0.34-1.26) | 0.82 (0.49-1.34) |
| **Age at diagnosis** |  |  |  |  |  |  |
| A1 <17 years | 2/62 (3.2%) | 11/89 (12.4%) | 1/40 (2.5%) | 0.074 | 1.000 | 0.233 |
|  |  |  |  | 4.23 (0.90-19.81) | 0.77 (0.07-8.77) | 3.08 (0.67-14.19) |
| A2 17-40 years | 51/62 (82.3%) | 62/89 (69.7%) | 29/40 (72.5%) | 0.089 | 0.324 | 0.111 |
|  |  |  |  | 0.49 (0.22-1.09) | 0.57 (0.22-1.47) | 0.52 (0.24-1.10) |
| A3 >40 years | 9/62 (14.5%) | 16/89 (17.9%) | 10/40 (25.0%) | 0.660 | 0.202 | 0.426 |
|  |  |  |  | 1.29 (0.53-3.14) | 1.96 (0.72-5.37) | 1.49 (0.65-3.40) |
| **Use of immuno-** | 45/61 (73.8%) | 68/90 (75.6%) | 32/41 (78.0%) | 0.850 | 0.648 | 0.721 |
| **suppressive agents**^1^ |  |  |  | 1.10 (0.52-2.32) | 1.26 (0.50-3.22) | 1.15 (0.57-2.31) |

Note: ^1^ Immunosuppressive agents included azathioprine, 6-mercaptopurine, and/or infliximab.
