# Supplementary material for: PTGER4 Expression-Modulating Polymorphisms in the 5p13.1 Region Predispose to Crohn's Disease and Affect NF-κB and XBP1 Binding Sites
Source: PLoS One. 2012 Dec 27;7(12):e52873. doi: 10.1371/journal.pone.0052873 (PMC3531335; doi:10.1371/journal.pone.0052873)
Supplement: Table S10 — Allele frequencies of the SNPs within the ATG16L1 gene in the North American replication cohort with Crohn's disease and controls. (DOC) [file pone.0052873.s010.doc]

**Supplementary Table S10. Allele frequencies of the SNPs within the *ATG16L1* gene in the North American replication cohort with Crohn’s disease and controls.**

| **Gene marker** | **Gene/region** | **Minor allele** | **Crohn’s disease** | | | **Controls** |
| --- | --- | --- | --- | --- | --- | --- |
|  |  |  | n=684 | | | n=1440 |
|  |  |  | **MAF** | **p value** | **OR [95 % CI]** | **MAF** |
| rs13412102 | *ATG16L1* | T | 0.34 | 3.28 x 10^-7^ | 0.71 [0.62-0.81] | 0.42 |
| rs3828309^+^ | *ATG16L1* | T | 0.39 | 3.60 x 10^-10^ | 0.66 [0.58-0.75] | 0.49 |
| rs2289474^+^ | *ATG16L1* | G | 0.39 | 2.97 x 10^-10^ | 0.66 [0.58-0.75] | 0.49 |
| rs2241880 (p.Thr300Ala)^+^ | *ATG16L1* | A | 0.39 | 3.71 x 10^-10^ | 0.66 [0.58-0.75] | 0.49 |
| rs2241879^+^ | *ATG16L1* | C | 0.39 | 3.71 x 10^-10^ | 0.66 [0.58-0.75] | 0.49 |

Note: Minor allele frequencies (MAF), allelic test p-values, and odds ratios (OR, shown for the minor allele) with 95% confidence intervals (CI) are shown. ^+^ surrogate markers for rs6431660.
