# Supplementary material for: PTGER4 Expression-Modulating Polymorphisms in the 5p13.1 Region Predispose to Crohn's Disease and Affect NF-κB and XBP1 Binding Sites
Source: PLoS One. 2012 Dec 27;7(12):e52873. doi: 10.1371/journal.pone.0052873 (PMC3531335; doi:10.1371/journal.pone.0052873)
Supplement: Table S11 — Allele frequencies of the SNPs within the ATG16L1 gene region in the German replication cohort with Crohn's disease patients and controls. (DOC) [file pone.0052873.s011.doc]

**Supplementary Table S11.** Allele frequencies of the SNPs within the *ATG16L1* gene region in the German replication cohort with Crohn’s disease patients and controls.

| **Gene marker** | **Gene/region** | **Minor allele** | **Crohn’s disease** | | | **Controls** |
| --- | --- | --- | --- | --- | --- | --- |
|  |  |  | n=1098 | | | n=1048 |
|  |  |  | **MAF** | **p value** | **OR [95 % CI]** | **MAF** |
| rs12471449 | *ATG16L1* | G | 0.10 | 7.51 x 10^-4^ | 0.73 [0.61-0.88] | 0.13 |
| rs6431660 | *ATG16L1* | A | 0.44 | 1.06 x 10^-5^ | 0.77 [0.68-0.86] | 0.47 |
| rs1441090 | *ATG16L1* | T | 0.06 | 3.37 x 10^-3^ | 0.68 [0.53-0.88] | 0.07 |
| rs2289472 | *ATG16L1* | G | 0.45 | 5.81 x 10^-6^ | 0.76 [0.67-0.86] | 0.47 |
| rs2241880 (p.Thr300Ala) | *ATG16L1* | T | 0.45 | 6.47 x 10^-3^ | 0.76 [0.67-0.86] | 0.47 |
| rs2241879 | *ATG16L1* | C | 0.45 | 9.47 x 10^-6^ | 0.76 [0.68-0.86] | 0.47 |
| rs3792106 | *ATG16L1* | A | 0.38 | 3.82 x 10^-5^ | 0.77 [0.69-0.87] | 0.41 |
| rs4663396 | *ATG16L1* | T | 0.17 | 1.72 x 10^-4^ | 0.74 [0.63-0.87] | 0.20 |

Note: Minor allele frequencies (MAF), allelic test p-values, and odds ratios (OR, shown for the minor allele) with 95% confidence intervals (CI) are shown.
