# Supplementary material for: PTGER4 Expression-Modulating Polymorphisms in the 5p13.1 Region Predispose to Crohn's Disease and Affect NF-κB and XBP1 Binding Sites
Source: PLoS One. 2012 Dec 27;7(12):e52873. doi: 10.1371/journal.pone.0052873 (PMC3531335; doi:10.1371/journal.pone.0052873)
Supplement: Table S12 — Epistasis analysis between SNPs rs4495224 and rs7720838 in the 5p13.1 region and SNPs within the ATG16L1 gene regarding CD susceptibility in the North American (NIDDK IBD Genetics Consortium) replication cohort. (DOC) [file pone.0052873.s012.doc]

**Supplementary Table S12.** Epistasis analysis between SNPs rs4495224 and rs7720838 in the *5p13.1* region and SNPs within the *ATG16L1* gene regarding CD susceptibility in the North American (NIDDK IBD Genetics Consortium) replication cohort.

|  | **5p13.1/*PTGER4* SNPs** | |
| --- | --- | --- |
| ***ATG16L1* SNPs** | **rs4532399^*^** | **rs7720838** |
|  | *P* value | *P* value |
| rs13412102 | 2.69 x 10^-1^ | 1.69 x 10^-1^ |
| rs2289474^+^ | 1.44 x 10^-1^ | 8.84 x 10^-2^ |
| rs2241880 (p.Thr300Ala) | 1.42 x 10^-1^ | 8.48 x 10^-2^ |

Note: ^+^ surrogate marker for rs6431660.
