# Supplementary material for: PTGER4 Expression-Modulating Polymorphisms in the 5p13.1 Region Predispose to Crohn's Disease and Affect NF-κB and XBP1 Binding Sites
Source: PLoS One. 2012 Dec 27;7(12):e52873. doi: 10.1371/journal.pone.0052873 (PMC3531335; doi:10.1371/journal.pone.0052873)
Supplement: Table S13 — Epistasis analysis between SNPs rs10941508 and rs7720838 in the 5p13.1 region and SNPs within the ATG16L1 gene regarding CD susceptibility in the German replication cohort. (DOC) [file pone.0052873.s013.doc]

**Supplementary Table S13. Epistasis analysis between SNPs rs10941508 and rs7720838 in the *5p13.1* region and SNPs within the *ATG16L1* gene regarding CD susceptibility in the German replication cohort.**

| ***ATG16L1* SNPs** | ***5p13.1*/*PTGER4* SNPs rs10941508^§^** |
| --- | --- |
|  | *P* value |
| rs13412102 | SNP not genotyped |
| rs12471449 | 7.85 x 10^-1^ |
| rs6431660 | 3.94 x 10^-1^ |
| rs1441090 | 8.84 x 10^-1^ |
| rs2289472 | 4.14 x 10^-1^ |
| rs2241880 (p.Thr300Ala) | 6.28 x 10^-1^ |
| rs2241879 | 3.88 x 10^-1^ |
| rs3792106 | 2.47 x 10^-1^ |
| rs4663396 | 8.97 x 10^-1^ |
